# Low Omega-3 intake is associated with high rates of depression and preterm birth on the country level

**DOI:** 10.1038/s41598-020-76552-x

**Published:** 2020-11-12

**Authors:** Timothy H. Ciesielski, Scott M. Williams

**Affiliations:** 1 Department of Population and Quantitative Health Sciences, Case Western Reserve University School of Medicine, 10900 Euclid Avenue, Cleveland, OH 44106 USA; 2 Ronin Institute, Montclair, NJ USA

**Keywords:** Epidemiology, Ecophysiology, Ecosystem services, Sustainability

## Abstract

Low circulating levels of long chain omega-3 polyunsaturated fatty acids (LC omega-3 PUFA) have been linked to major depressive disorder (MDD) and preterm birth (PTB), and prenatal depression associates with PTB. We therefore hypothesized that low Omega-3 intake would associate with higher MDD and PTB rates on the country-level. To test this hypothesis, we obtained country-level estimates for omega-3 intake, MDD prevalence, PTB rate, and per capita income for 184 countries in 2010. We then estimated the LC omega-3 PUFA levels that these intakes produce by accounting for direct consumption and the endogenous conversion of ingested plant-based precursors. Penalized splines indicated that MDD and PTB rates decreased linearly with increasing LC omega-3 PUFA, up to ~ 1000 mg/day for MDD and up to ~ 550 mg/day for PTB. Adjusted linear regression models below these thresholds revealed that a one standard deviation increase in LC omega-3 PUFA (380 mg/day) was associated with an MDD decrease of 5 cases/1000 people and a PTB decrease of 15 cases/1000 livebirths. In light of the extensive prior evidence on the individual-level, these findings indicate that low intake of LC omega-3 PUFA and its precursors may be elevating MDD and PTB rates in 85% of the countries studied.

## Introduction

Women with prenatal depression are at increased risk of having a preterm birth (PTB)^1–4^, but unfortunately intra-pregnancy antidepressant medications do not reduce the risk, and they may actually increase the risk of preterm birth and other adverse pregnancy outcomes^5–10^. Overall, these two conditions, prenatal depression and preterm birth, merit substantial research effort because they are highly prevalent and consequential. Worldwide, prenatal depression occurs in 7–25% of pregnancies^11^, while preterm delivery is present in 5–18% of livebirths^12^, and both are linked to serious and long-term health problems in the offspring^13–18^. Fortunately, a variety of non-pharmacologic factors may reduce the risk of depression, and supplementation with Long Chain Omega3 Polyunsaturated Fatty Acids (LC Omega3 PUFA) has demonstrated efficacy against Major Depressive Disorder (MDD) in some contexts^19–21^. However, given the complexities of LC omega3 PUFA research^22^, including the necessity of considering baseline intakes, sufficiency thresholds, and the endogenous conversion of ingested precursors, the findings have been inconsistent^23,24^. In short, it is not always clear who will have mood improvements in response to omega3 interventions of various types. Importantly, there is also strong meta-analytic evidence that prenatal omega-3 supplementation can reduce the risk of preterm birth^25^, but this literature has been plagued with similar complexities^26,27^. In some circumstances intra-pregnancy supplement pills fail to reduce the risk of preterm birth, and in others they may increase the risk of post-term delivery^28–30^.

Overall, the evidence from clinical trials includes mixed findings for both outcomes, but the results indicate that omega-3 supplementation can, on average, reduce depressive symptomology and the risk of preterm birth^23,25^. However, at this point, we cannot characterize the clinical efficacy in specific settings, because none of the 30+ MDD trials^23^ or 70+ PTB trials^25^ concurrently accounted for all 3 critical factors: baseline intakes, sufficiency thresholds, and the endogenous conversion of ingested precursors. Given these possible weaknesses in the clinical trial evidence, it is critical to consider the scientific literature beyond the RCTs of Omega-3 PUFA supplements. While the quality and efficacy of Omega-3 PUFA supplements, and their epidemiologic assessment has varied, the underlying physiologic findings are more definitive. Low levels of LC Omega-3 PUFA are associated with both depression^31–35^ and prenatal depression^36^, and at mid-pregnancy, low levels predict preterm birth^37,38^. This begs the question: Are supplement pills the best way to achieve appropriate internal levels of LC Omega-3 PUFA? Evidence from a wide variety of observational epidemiology studies and the re-evaluation of baseline Omega-3 PUFA blood levels from a recent RCT indicate that high habitual intake of Omega-3 PUFAs may be associated with reduced risk of depression^39^, perinatal depression^40^, and preterm birth^22,26,41,42^. If this is the case, it is possible that diets with low Omega-3 PUFA content may simultaneously increase the risk of depression and preterm birth.

While most of the epidemiologic studies in this area have not addressed potential mechanisms, a growing pile of molecular evidence indicates that this hypothesis is biologically plausible. With regard to MDD, low Omega-3 PUFA levels can alter serotonin, dopamine, glucocorticoid, lipid receptor, and endocannabinoid mediated signaling as well as exacerbate neuro-inflammation and impair neurogenesis^43–45^. With respect to PTB, low Omega-3 levels can increase the production of utero-tonic (labor inducing) prostaglandins, promote cell death in placental trophoblasts, and have a wide variety of deleterious perinatal inflammatory effects^46–48^.

All of this evidence in aggregate, has led us to hypothesize that communities with low Omega-3 PUFA intakes will have high rates of both depression and preterm birth. In this study, we test this hypothesis in available country-level data from 2010. This approach cannot estimate intake requirements on the level of the individual but it will allow us to detect the presence of sufficiency thresholds in country intake estimates. Furthermore, this method will allow us to consider the role of food systems, and explore the possibility that low Omega-3 intake may increase country-level rates of expensive and often devastating pathologies^12,49–55^. Finally, this analysis will allow us to estimate the global scale of LC Omega-3 PUFA deficiency and two of its critical consequences.

## Methods

We obtained published country-level estimates for MDD prevalences^52^, Omega-3 intakes^56,57^, PTB rates^12^ and country income levels^12^ for 184 countries in 2010. These countries were selected because they had information on the four variables of interest.

### MDD data

Country-level estimates of age-standardized MDD prevalence (cases per 100 people) were generated by Ferrari et al.^52^ as part of the 2010 Global Burden of Diseases, Injuries, and Risk Factors Study. The authors have described their approach in detail^52,58,59^, but we provide a summary here. In short, the authors conducted a systematic literature review to obtain published MDD prevalence estimates from around the world from January 1st 1980 until December 31st 2008 (and they note that they continued to peruse the literature until 2011). They then used a Bayesian meta-regression tool (DisMod-MR) to estimate harmonized point prevalences for MDD in 2010 while accounting for differences in data types, uncertainty levels, and missing data. MDD was defined according to the description of recurrent depressive disorder in ICD-10: The presence of at least one major depressive episode, which involves the experience of depressed mood for the majority of the day, every day, for 14 consecutive days^52,60^. MDD prevalence estimates include both male and female cases.

### Omega-3 and PTB data

Initial collection of the Omega-3 and PTB data has been previously described^12,56,61,62^ and was also summarized in our prior publication^22^. In brief, the Omega-3 intakes were estimated by the Nutrition and Chronic Diseases Expert Group (NutriCoDE) for the 2010 Global Burden of Diseases, Injuries, and Risk Factors Study^56,62^. The NutriCoDE group used a Bayesian hierarchical imputation model to generate harmonized estimates of fatty-acid intakes that account for the heterogeneity of the source data. The PTB rates were also estimated from a group of heterogeneous datasets, and in this case the authors used regression models to harmonize the estimates across the distinct data qualities in the developed and developing world^12^. PTB was defined as gestation lasting less than 37 weeks.

### Country income data

Limited covariate information was available to evaluate for potential confounding. However Blencowe et al.^12^ provided information on one important variable: mean country income in 2010. For each of the 184 countries, they provided a four category ordinal ranking based on per capita Gross National Income (World Bank Atlas Method). In this WHO metric a higher rank corresponds to a higher mean income for the residents of that country.

### Calculating the composite omega-3 metric

Micha et al.^56,62^ provided mean intakes for plant-based and seafood-based Omega3 PUFA. These intakes were estimated in mg/day and they were based only on diet (potential intake from supplements was not included). Humans can convert plant based (alpha-linolenic acid, ALA) into LC Omega-3 PUFA (eicosapentaenoic acid, EPA and docosahexaenoic acid, DHA) and thus estimates of LC Omega-3 PUFA exposure that do not account for this conversion are incomplete^63–65^. Because women can convert about 21% of ingested ALA into EPA, and men can convert about 8%, the global population likely converts, on average, around 15% of ingested ALA into EPA^63–65^. We included both sexes in our analyses and thus we estimated LC Omega-3 PUFA (mg/day) for each country as follows:

$$ Total\,LC\,Omega - 3 PUFA = seafood\,based + \left(plant\,based\,\times 0.15 \right) $$

Here we define LC Omega-3 PUFA intake as the seafood-based intake (EPA and DHA) plus 15% of the plant-based intake (ALA), which we estimate was converted endogenously. This is the most empirically supported conversion rate average, but because these rates may vary by nutritional^66,67^ and genetic factors^68,69^ that we cannot evaluate here, we performed supplementary analyses that utilized a variety of alternative conversion rates. To assess the possibility that plant and seafood-based intakes might have distinct effects on these outcomes, we also conducted supplementary analyses that evaluated these intakes as separate independent variables.

### Statistical analysis

Previous analyses suggest that the relationship between LC Omega-3 PUFA levels and PTB is nonlinear^22,37^. PTB risk decreases with increasing LC Omega-3 PUFA levels, but once sufficiency is achieved, PTB risk becomes independent of Omega3 intake; this pattern has been observed in analyses on both the individual and country level^22,37^. Importantly, most of the early perinatal research on LC Omega-3 PUFA did not consider the existence of a threshold, and this may account for some of the heterogeneity seen in the literature. Because there may be a similar threshold in the relationship between LC Omega-3 PUFA and MDD prevalence, we evaluated this relationship with penalized splines. This approach models MDD prevalence as the outcome and considers LC Omega-3 PUFA as a smoothed exposure variable (a spline). We used GAM from the mgcv package in R (v3.5.0) to implement a generalized cross validation procedure (GCV), which provides a standard iterative process for identifying evidence of nonlinearities^70,71^. If there is sufficient evidence for a threshold, then GCV provides a smoothed piecewise function to model the relationship between the variables. Otherwise, GCV identifies a single slope throughout the exposure distribution and returns a linear term. Because the GCV process returns a spline with evidence of a threshold at ~ 1000 mg/day and the relationship appears linear on either side of this value, we constructed separate linear regression models in these two sections of the exposure distribution. As noted above, we had limited covariate information but we were able to construct models adjusted for country-level income and PTB rate. We also re-assessed the omega3-PTB relationship^22^ in a similar fashion. We obtained splines, observed a putative threshold, fit linear regression models in the two linear regions of the relationship, and we were able to add new adjustments for MDD prevalence.

As in our initial PTB analyses^22^, we were not attempting to characterize the relationship between LC Omega-3 PUFA and MDD on the individual-level. We were also not attempting to determine what intake or internal levels would be sufficient for an individual. These analyses simply assess if low Omega-3 intake is associated with elevated MDD prevalence on the country-level. Penalized spline and regression analyses were performed with R 3.5.0 (https://www.r-project.org/), and other analyses were conducted with SAS 9.4 (SAS Institute, Cary NC).

## Results

There were 184 countries with complete data on MDD prevalence, Omega-3 intake, PTB rate, and country income level (Table 1 and Table S1). Among these countries the mean prevalence of MDD was 5.3 cases per 100 people (SD: 1.8) and the mean LC Omega-3 PUFA was 343 mg/day (SD: 380). The mean PTB rate was 10.2 PTBs per 100 live births (SD: 3.0). The two covariates, country income and PTB rate, were correlated with LC Omega-3 PUFA (Spearman rank correlations of 0.44 and -0.32 respectively) and these correlations were unchanged when the Maldives was excluded (LC omega-3 PUFA outlier; Figs.S1 and S2).

**Table 1:** Descriptive statistics for the 184 countries.

	Median	Mean	IQR	Standard deviation	Range
All Countries (n = 184)					
Prevalence of MDD (cases per 100 people)	5.1	5.3	4.4, 6.0	1.8	2.5, 22.5
Total LC Omega-3 PUFA (mg/day)^a^	262	343	155, 404	380	35, 3918
Gross national income^b^	2.0	1.6	1.0, 2.5	1.1	0.0, 3.0
PTB rate (PTBs per 100 live births)	10.2	10.2	7.7, 12.4	3.0	4.1, 18.1
Countries excluding outliers^c^ (n = 182)					
Prevalence of MDD (cases per 100 people)	5.1	5.2	4.4, 6.0	1.3	2.5, 9.3
Total LC omega-3 PUFA (mg/day)^b^	262	324	156, 403	273	35, 2011
Gross national income^c^	2.0	1.6	1.0, 3.0	1.1	0.0, 3.0
PTB rate (PTBs per 100 live births)	10.2	10.2	7.6, 12.4	3.0	4.1, 18.1

^a^LC Omega-3 PUFA = seafood based intake + (plant based intake × 0.15).

^b^Gross National Income coded as a variable with 4 levels using the World Bank Atlas Method (0 = low income, 3 = high income).

^c^Two countries were excluded as outliers: Afghanistan (MDD prevalence outlier: 22.5 cases per 100 people; over 9 SD from the global mean) and the Maldives (LC Omega-3 PUFA outlier: 3918 mg/day; over 9 SD from the global mean).

**Figure 1:**
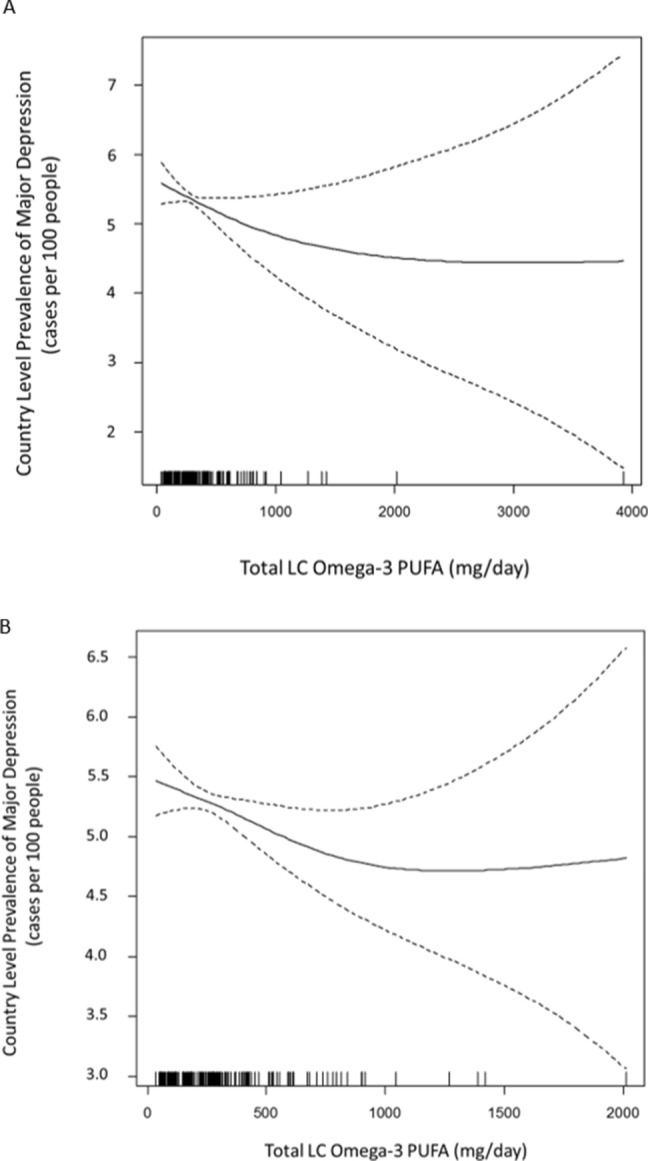
Penalized spline showing the relationship between LC Omega-3 PUFA intake and MDD prevalence. LC Omega-3 PUFA = seafood based intake + (plant based intake × 0.15) (**A**) Country-level LC Omega-3 PUFA intakes are on the x-axis and country-level prevalences of MDD are on the y-axis. Each vertical line on the x-axis denotes a single country and this allows the figure to depict the data density in different regions of the exposure distribution. (**B**) Same as panel A, except two countries were excluded as outliers (n = 182); Afghanistan was excluded as an MDD outlier (22.5 cases per 100 people; over 9 SD from the global mean) and the Maldives was excluded as an LC Omega-3 PUFA outlier (3918 mg/day; over 9 SD from the global mean). Figure made with R 3.5.0 (https://www.r-project.org/).

The GCV procedure in the spline modeling process identified evidence of non-linearity in the Omega-3-MDD relationship whether two outliers were excluded or not (Fig. 1). Both of these penalized splines indicate the presence of a threshold between 900 and 1100 mg/day of LC Omega-3 PUFA. The omega3-MDD relationship appears linear on either side of 1000 mg/day, and thus separate linear regression analyses (Table 2) were conducted above and below this putative threshold. The unadjusted model for countries below 1000 mg/day (n = 177) indicates that MDD prevalence decreases by 0.46 cases per 100 people (95% CI 0.10, 0.82) for each 1 SD increase in LC Omega-3 PUFA intake (380 mg/day). After adjusting for country income level and PTB rate the association was similar (0.50 cases per 100 people, 95% CI 0.13, 0.88). Country income and PTB rate were positively associated with MDD prevalence (Table 2). There was no significant association between LC Omega-3 PUFA and MDD prevalence above the 1000 mg/day threshold (Table 2).

**Table 2:** Results from linear regression models within the 2 sections of the omega3-MDD relationship^a^.

	Change in prevalence of depression (cases per 100 people)	95% CI	*p* value
< 1000 mg/day LC Omega-3 PUFA (n = 177)			
Model 1: Unadjusted			
LC Omega-3 PUFA^b^	− 0.457	− 0.816, − 0.097	0.014
Model 2: Income adjusted			
LC Omega-3 PUFA^b^	− 0.553	− 0.932, − 0.173	0.005
Country income^c^	0.140	− 0.044, 0.325	0.137
Model 3: Income and PTB adjusted			
LC Omega-3 PUFA^b^	− 0.504	− 0.881, − 0.127	0.010
Country income^c^	0.251	0.046, 0.455	0.017
Preterm birth rate^d^	0.083	0.013, 0.153	0.021
≥ 1000 mg/day LC Omega-3 PUFA (n = 5)			
Model 1: Unadjusted			
LC Omega-3 PUFA^b^	− 0.035	− 0.403, 0.332	0.863
Model 2: Income adjusted			
LC Omega-3 PUFA^b^	0.102	− 0.295, 0.500	0.664
Country income^c^	− 0.441	− 1.124, 0.242	0.333
Model 3: Income and PTB adjusted			
LC Omega-3 PUFA^b^	− 0.212	− 0.466, 0.042	0.349
Country income^c^	1.409	0.248, 2.570	0.254
Preterm birth rate^d^	0.340	0.133, 0.547	0.192

^a^LC Omega-3 PUFA = seafood based intake + (plant based intake × 0.15) Two countries were excluded as outliers: Afghanistan (MDD prevalence outlier: 22.5 cases per 100 people; over 9 SD from the global mean) and the Maldives (LC Omega-3 PUFA outlier: 3918 mg/day; over 9 SD from the global mean).

^b^Change in the prevalence of MDD (cases per 100 people) with a 1 SD increase in LC Omega-3 PUFA intake (380 mg/day).

^c^Change in the prevalence of MDD (cases per 100 people) associated with a 1 unit increase in GNI (a rank variable with four levels; higher rank corresponds to higher income).

^d^Change in the prevalence of MDD (cases per 100 people) associated with a 1 unit increase in PTB Rate (cases per 100 live births).

**Figure 2:**
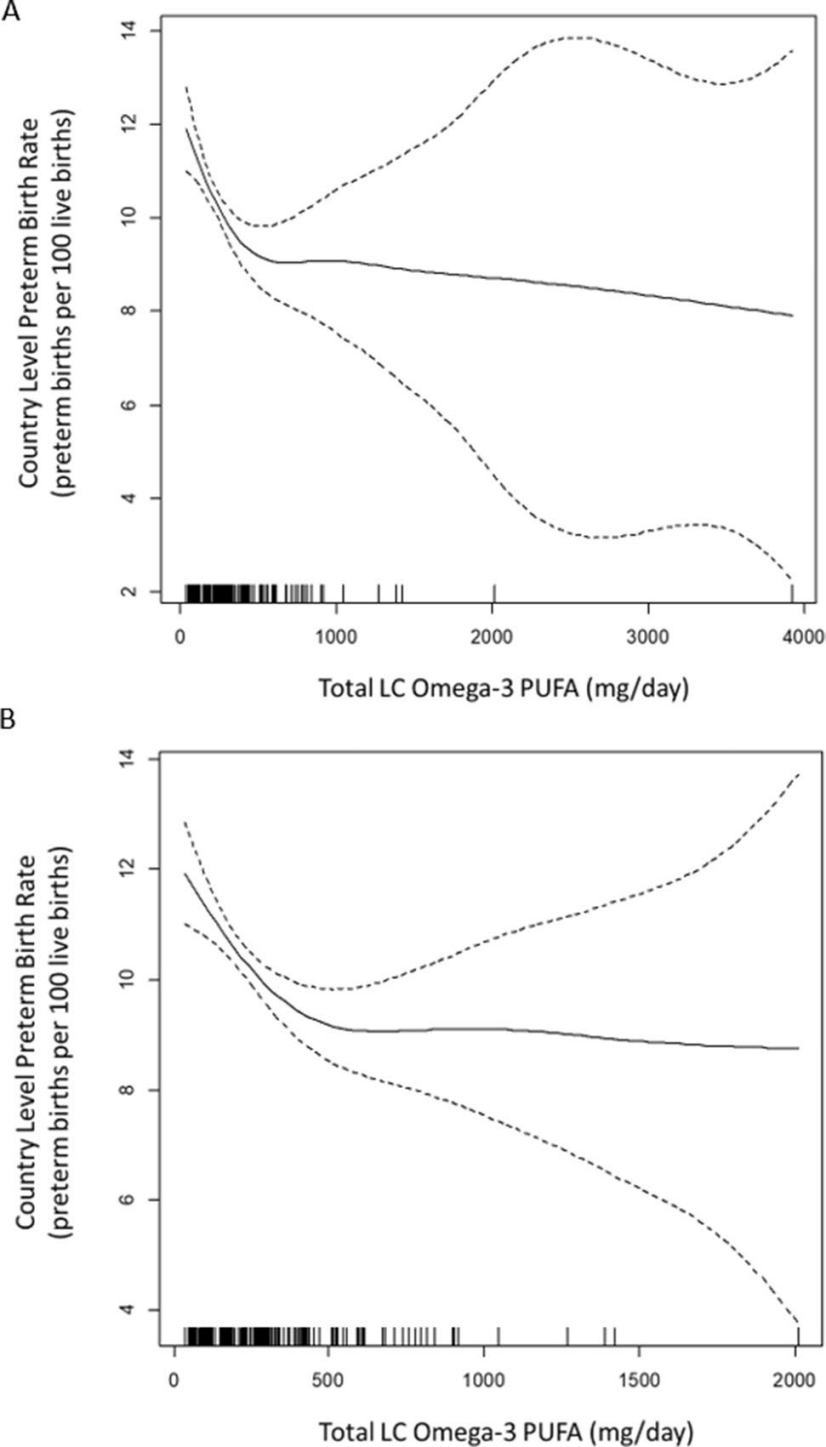
Penalized spline showing the relationship between LC Omega-3 PUFA intake and PTB rate. LC Omega-3 PUFA = seafood based intake + (plant based intake × 0.15) (**A**) Country-level LC Omega-3 PUFA intakes are on the x-axis and country-level PTB rates are on the y-axis. Each vertical line on the x-axis denotes a single country and this allows the figure to depict the data density in different regions of the exposure distribution. (**B**) Same as panel A, except two countries were excluded as outliers (n = 182): Afghanistan (MDD prevalence outlier: 22.5 cases per 100 people; over 9 SD from the global mean) and the Maldives (LC Omega-3 PUFA outlier: 3918 mg/day; over 9 SD from the global mean). Figure made with R 3.5.0 (https://www.r-project.org/).

We also re-evaluated our previous PTB analyses^22^ with the *sex-averaged* ALA to EPA conversion rate (15%), and adjusted for MDD prevalence (Fig. 2 and Table 3). In the penalized spline analyses a putative threshold was identified between 500 and 600 mg/day (Fig. 2). The unadjusted linear regression model for countries below the 550 mg/day threshold (n = 157) indicates that PTB rate decreases by 3.1 cases per 100 live births (95% CI 1.7, 4.5) for each 1 SD increase in LC Omega-3 PUFA intake (380 mg/day). After adjusting for country income and MDD prevalence the association was smaller in magnitude but still significant (1.5 cases per 100 live births, 95% CI 0.2, 2.9). Country income was negatively associated with PTB rate and MDD prevalence was positively associated with PTB rate (Table 3). There was no significant association between LC Omega-3 PUFA and PTB rate above the 550 mg/day threshold (Table 3).

**Table 3:** Results from linear regression models within the 2 sections of the omega-3-PTB relationship^a^.

	Change in the PTB rate (cases per 100 live births)	95% CI	*p* value
< 550 mg/day LC Omega-3 PUFA (n = 157)			
Model 1: Unadjusted			
LC Omega-3 PUFA^b^	− 3.09	− 4.47, − 1.71	2.1 × 10^–5^
Model 2: Income adjusted			
LC Omega-3 PUFA^b^	− 1.69	− 3.06, − 0.32	1.7 × 10^–2^
Country Income^c^	− 1.15	− 1.58, − 0.72	4.0 × 10^–7^
Model 3: Income and MDD adjusted			
LC Omega-3 PUFA^b^	− 1.51	− 2.88, − 0.15	3.2 × 10^–2^
Country income^c^	− 1.21	− 1.64, − 0.79	9.7 × 10^–8^
MDD prevalence^d^	0.36	0.03, 0.68	3.4 × 10^–2^
≥ 550 mg/day LC Omega-3 PUFA (n = 25)			
Model 1: Unadjusted			
LC Omega-3 PUFA^b^	− 0.41	− 1.71, 0.90	5.5 × 10^–1^
Model 2: Income adjusted			
LC Omega-3 PUFA^b^	0.44	− 0.59, 1.46	4.1 × 10^–1^
Country income^c^	− 2.13	− 3.05, − 1.22	1.5 × 10^–4^
Model 3: Income and MDD adjusted			
LC Omega-3 PUFA ^b^	0.33	− 0.72, 1.38	5.5 × 10^–1^
Country income ^c^	− 2.02	− 2.97, − 1.07	4.3 × 10^–4^
MDD prevalence ^d^	0.45	− 0.45, 1.34	3.4 × 10^–1^

^a^LC Omega-3 PUFA = seafood based intake + (plant based intake × 0.15) Two countries were excluded as outliers: Afghanistan (MDD prevalence outlier: 22.5 cases per 100 people; over 9 SD from the global mean) and the Maldives (LC Omega-3 PUFA outlier: 3918 mg/day; over 9 SD from the global mean).

^b^Change in the PTB rate (cases per 100 live births) associated with a 1 SD increase in LC Omega-3 PUFA intake (380 mg/day).

^c^Change in the PTB rate (cases per 100 live births) associated with a 1 unit increase in GNI (a rank variable with four levels; higher rank corresponds to higher income).

^d^Change in the PTB rate (cases per 100 live births) associated with a 1 unit increase of MDD (cases per 100 people).

**Figure 3:**
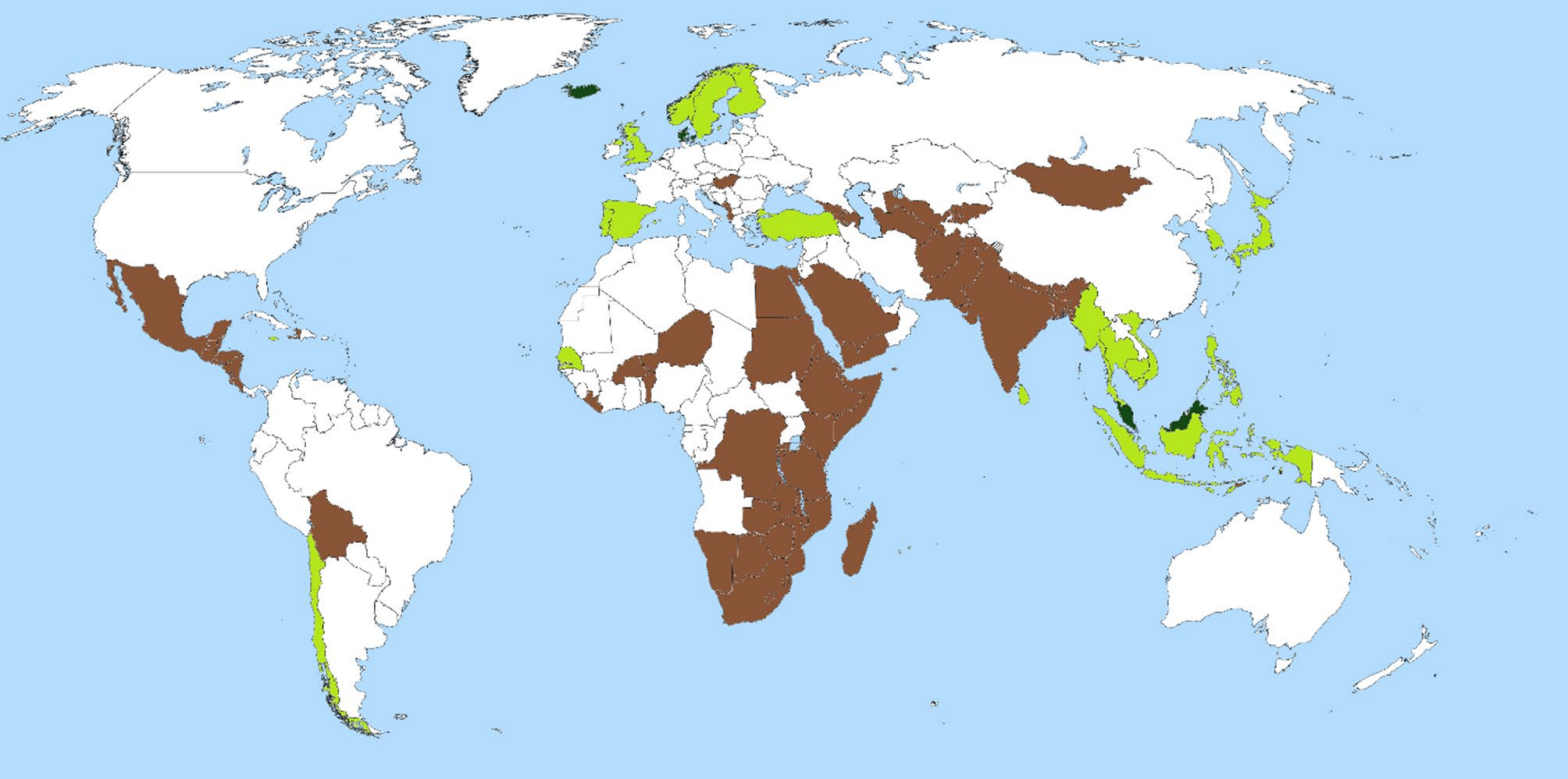
Countries with low and high Omega-3 intake among adults. LC Omega-3 PUFA = seafood based intake + (plant based intake × 0.15) Countries in dark green have LC Omega-3 PUFA intakes that are above the putative sufficiency threshold for MDD (> 1000 mg/day, n = 6 when the Maldives are included). Countries in light green have LC Omega-3 PUFA intakes that are above the putative sufficiency threshold for PTB (> 550 mg/day, n = 26 when the Maldives are included). All other countries are below both thresholds (< 550 mg/day, n = 158 when Afghanistan is included). Countries in brown (n = 53) have LC Omega-3 PUFA intakes < 170 mg/day, and this is at least one standard deviation (380 mg/day) below the PTB sufficiency threshold (550 mg/day). Country names and LC Omega-3 PUFA intakes are listed in Table S1. Adapted from a map image that was released into the public domain by the author Petr Dlouhy (Source: https://commons.wikimedia.org/wiki/File:A_large_blank_world_map_with_oceans _marked_in_blue.svg#file).

There were clear geographic patterns in the distribution of LC Omega-3 PUFA and countries with low intake were primarily found in Africa, South-Central Asia, and Central America (Fig. 3). Countries with higher Omega-3 intake were primarily located in Southeast Asia and along the North Atlantic coast of Europe. Six countries had LC Omega-3 PUFA intakes above 1000 mg/day and 26 countries were above 550 mg/day. 158 of the 184 countries had LC Omega-3 PUFA intakes below 550 mg/day.

When seafood and plant based omega-3 PUFA were analyzed separately in the PTB analysis, the spline for seafood based omega-3 PUFA indicated a threshold at 400–550 mg/day and the spline for plant based omega-3 PUFA indicated a threshold at 2500–3500 mg/day (Fig. S3). In the MDD analysis the spline for seafood based omega-3 PUFA indicated a threshold at 850–1100 mg/day and for plant based Omega-3 PUFA no threshold was detected within the range of observed exposures (Fig. S3). In both the PTB and MDD analyses, the shape of the splines was similar when the assumed conversion rate for plant based precursors was varied between 0 and 25% (Figs. S4 and S5).

## Discussion

We observed that country-level MDD prevalence decreases as LC Omega-3 PUFA intake increases. Additionally, we found that this relationship has a threshold, and increases above ~ 1000 mg/day are not associated with additional changes in MDD prevalence. Perhaps most importantly these conclusions remained after adjusting for country income and PTB rates. Because numerous causal structures can be posited to describe the relationships between these 3 variables it is unclear if these adjustments serve to decrease or increase bias in the association estimate (Fig. S6). However, the association is similar and significant in the unadjusted and adjusted models, and thus bias from these variables does not explain the observed association. We also found that PTB rates decrease with increasing LC Omega-3 PUFA up to ~ 550 mg/day, and though the magnitude of the association is reduced after adjustment for country income and MDD prevalence, potential bias from these sources does not explain the observed relationships. Finally, both analyses indicated that MDD prevalences and PTB rates are positively correlated on the country-level, which is consistent with previous studies on the individual-level (ours and others) linking adverse pregnancy outcomes to depression^1–4,72,73^.

### Depression

Our findings linking dietary Omega-3 and MDD on the country-level align very well with meta-analytic findings on the individual-level^39^. Grosso et al. examined 31 studies that included over 250,000 individuals; they reported a significant inverse association between depression and habitual LC Omega3 intake (EPA + DHA) as well as total Omega-3 intake (ALA + EPA + DHA). Furthermore, their dose–response analyses indicated the presence of sufficiency thresholds. Although our analysis was not intended to detect a sufficiency threshold on the level of the individual, the country-level threshold in our analysis is consistent with their individual-level findings^39^. In short, their EPA + DHA analysis effectively assumed a 0% conversion of ALA to EPA, thus these exposure estimates were low and they resulted in a low threshold (200–300 mg/day). On the other hand, their total Omega-3 intake analysis effectively assumed a 100% conversion of ALA to EPA, thus these exposure estimates were high and they resulted in a high threshold (around 1800 mg). Our analysis utilized an empirically supported intermediate conversion rate (15%) and identified a threshold between their two values (~ 1000 mg).

Individual-level RCTs have also been used to evaluate the efficacy of LC Omega-3 PUFA supplements in the treatment of depression. The results of these studies have been mostly positive, but there were some inconsistencies^19,20,23,74,75^. However, some of these studies have features that limit their interpretability and none of them were intended to address the hypothesis that we were testing (with regard to chronic dietary intake). In short, these RCTs often failed to appropriately account for baseline intakes, the existence of a sufficiency threshold, and the conversion of ingested ALA to EPA. Despite these complexities and the heterogeneity they produce, a recent meta-analysis concluded that Omega-3 supplements had significant therapeutic effects in specific contexts^19^. Overall, the RCTs attempted to test the efficacy of Omega-3 supplement pills for treatment, and they were not intended to characterize the role of habitual food-based intake in the prevention of depression.

Research on internal levels of LC Omega-3 PUFA can provide critical convergent evidence in this context, because this approach has complementary strengths^76^ when compared to supplement or diet-based studies. Blood-based studies are not prone to the exposure estimation weaknesses seen in RCTs (difficulty in accounting for baseline intakes and ALA conversion rates), and population based studies of habitual intake (difficulty in accounting for ALA conversion rates). Importantly, these internal biomarker studies have found that low internal levels of LC omega3 PUFA are strongly associated with depression^31–35^ and prenatal depression^36^. Furthermore, there is growing evidence that people who respond to LC Omega-3 PUFA interventions with reduced depressive symptoms are those who experience elevations in internal LC Omega-3 PUFA levels^21,77,78^. Overall, the blood-based studies provide a more precise characterization of insufficiency, and the intake studies are starting to clarify the dietary patterns that can prevent insufficiency and its affective consequences.

### Preterm birth

We previously reported a link between LC Omega-3 PUFA and PTB rate on the country-level^22^, and we now extend these findings with additional consideration of covariates and modeling assumptions. Our previous analysis utilized female specific Omega-3 intakes and precursor conversion rates. In this new analysis we consider country-level MDD prevalence, and because MDD occurs in both males and females, we utilized sex-averaged intake estimates and conversion rates. Because males tend to have lower ALA to EPA conversion rates, the mean conversion rate was reduced from 20 to 15%^63–65^. This subtle decrease in exposure estimates resulted in the detection of a slightly lower sufficiency threshold (~ 550 mg/day as opposed to ~ 600 mg/day), but otherwise the shape of the penalized spline was very similar. For countries below this putative sufficiency threshold we observed that LC Omega-3 PUFA intake is inversely associated with PTB rates. This conclusion remained unchanged after adjusting for country income and MDD prevalence, indicating that potential bias from these two variables does not fully explain the association. Finally, we note that these PTB findings on the country-level are corroborated by the findings on the individual-level. Circulating maternal LC Omega-3 PUFA levels in pregnancy predict shorter gestational length^79^ and PTB^37,38^, and increasing LC Omega-3 PUFA intake in pregnancy can lower the risk of PTB^25^ in some settings. Future intake studies that consider baseline intakes, sufficiency thresholds, and the endogenous conversion of ingested precursors could clarify who needs to increase their LC Omega-3 PUFA levels and how they can do this safely.

### Endogenous ALA to EPA conversion rates

We are aware that ALA to EPA conversion rates can vary by genetic^68,69^ and nutritional^66,67^ factors that we do not evaluate here, but 15% is the most empirically supported conversion rate average for joint-sex analyses^63–65^. Furthermore, this rate is consistent with the patterns we observed when seafood and plant based Omega-3 PUFA were analyzed separately (Fig. S3). When we considered alternative mean conversion rates we obtained qualitatively similar splines, and detected subtle, yet predictable, shifts in the locations of the thresholds (Figs. S4 and S5). Overall, this re-affirms the existence of sufficiency thresholds in country-level Omega-3 intakes, and indicates that they cannot be precisely determined in the absence of empirically derived country-level conversion rates. This problem is not unique to the country-level, and in the absence of blood measurements, the precise determination of intake sufficiency in individuals would also require personalized conversion rate information. Overall, studies which measure internal LC Omega-3 PUFA levels may be able to estimate sufficiency levels more precisely than studies that depend on intake data alone.

### Putative mechanisms

The epidemiologic studies linking LC Omega-3 PUFA, depression, and PTB have generally not evaluated potential mechanisms. However, molecular evidence is starting to clarify why LC Omega-3 PUFA deficiency could increase the likelihood of both depression and PTB. For MDD, it appears that insufficient Omega-3 levels can: (1) alter hormonal signaling and neurotransmitter function (e.g. serotonin, dopamine, glucocorticoid, lipid receptor, and endocannabinoid mediated signaling), (2) increase neuro-inflammatory processes, and (3) impair neurogenesis^43–45^. For PTB, insufficient Omega-3 levels can: (1) have broad inflammatory effects, (2) increase placental trophoblast cell death, and (2) intensify the biochemical conversion of omega-6 PUFA into utero-tonic (labor inducing) prostaglandins^46–48^. Although the mechanisms are not yet clear, this molecular evidence indicates that MDD and PTB could be linked, in part, because LC Omega-3 PUFA deficiency creates physiologic conditions that increase the risk of both of them (i.e. nutritional pleiotropy).

### What is a sufficient country-level intake? Who has achieved sufficiency?

Six countries are above the putative sufficiency threshold for MDD (> 1000 mg/day) and 26 countries are above the putative sufficiency threshold for PTB (> 550 mg/day). This indicates that all of the 158 remaining countries are below both thresholds (< 550 mg/day), and the regression models predict that they could decrease both PTB and MDD by increasing their Omega-3 intake. 53 countries have LC Omega-3 PUFA intakes that are at least one standard deviation below the PTB sufficiency threshold (< 170 mg/day). The adjusted regression model estimates that raising LC Omega-3 PUFA in these countries to 550 mg/day could reduce PTB rates by at least 15 cases per 1000 births. Additionally, this change would be expected to decrease MDD prevalence by at least 5 cases per 1000 people. Raising LC Omega-3 PUFA intake in these countries to 1000 mg/day would be expected to generate the same reduction in PTB rates (due to going above the threshold), but a larger reduction in MDD prevalence (a decrease of at least 11 cases per 1000 people). Thus, assuming mean baseline rates of PTB and MDD, an increase to 1000 mg/day would be expected to reduce PTB rate by ~ 15% and MDD prevalence by ~ 22%. Finally, we note that while blood level screening cannot remedy a chronic food system inadequacy on the country-level, defining sufficiency levels for erythrocyte Omega-3 content on the individual-level remains a critical goal for clinical management^80^. Obviously, the development of MDD and PTB depends on many factors, but LC Omega-3 PUFA sufficiency could remove a component cause^81^ and lower the probability of these diseases.

### Strengths and weaknesses of this analysis

Given the country-level nature of our analysis, we had limited access to covariate information and our study, like all observational epidemiology studies, is prone to potential bias from variables that are relevant, but not measured. In general, factors that associate with exposure (LC Omega-3 PUFA), and outcome (MDD or PTB) and are not mediators^82,83^ could potentially generate bias in our association estimates. We would want to assess these variables with directed acyclic graphs to determine to determine an adjustment strategy that reflects our current understanding of the causal structures^82,83^. In this light, we would have liked to assess country-level information on other nutrients that could alter the risk of PTB^84–86^ or depression^87,88^ or both (e.g. folate^89,90^ and vitamin D^85,91^). Country-level information on the number of previous births or preterm births was not available, but we note that these variables might be inappropriate to adjust for^92,93^ even if they were available, as we do not have a priori expectations on how these could affect the results. We also could not evaluate intrapregnancy changes in PUFA levels or placental transfer mechanisms^94^, or assess the genetic, metabolic, or lifestyle factors that may associate with maternal blood levels of LC Omega-3 PUFA (e.g. FADS genotype, diabetes, alcohol intake, smoking, and LC Omega-3 PUFA supplement use^95–97^).

While we did not have country-level information on Omega-3 PUFA supplement pill use, we note that high country income would be a proxy for the availability of these supplements, and adjustment for country income does not change our conclusions in either the MDD or PTB analysis. We have no data on how women in different countries might increase or decrease their seafood intake when pregnant, but transient changes in diet would be expected to have little effect on the average habitual intake. Furthermore, these changes would not influence the long-term pre-pregnancy diet which appears to be most critical here^42^. Having said this, in order to generate bias in our PTB analysis, women in countries with low PTB rates would have to change their seafood intake in pregnancy to a different extent than women in countries with high PTB rates. Lastly, our analyses focused on intake and could not directly identify relationships between circulating levels of Omega-3 PUFA and clinical variables.

We also acknowledge that we cannot rule out the possibility of reverse causation (due to the cross-sectional nature of our analysis), or separately evaluate distinct subtypes of LC-Omega-3 PUFA (i.e. EPA vs. DHA) in our country-level analysis. Finally, we note that depression and PTB are complex conditions and here we have assessed the role of just one of many potential causal components. Other factors associated with MDD may cause PTB and other factors associated with PTB may cause MDD, but the associations with LC Omega-3 PUFA intake remain significant in the PTB and MDD adjusted models. Thus, we find no evidence that these conditions and presumably their unmeasured, underlying risk factors explain the associations with LC Omega-3 PUFA.

In this study our hypotheses, data, analyses, observations, and inferences were all on the country-level. Nonetheless, we made these group-level hypotheses because a wide variety of prior studies on the individual-level (laboratory, observational epidemiology, and RCT findings) indicate that low Omega-3 intake can cause depression^39,78,91,98^ and PTB^25,37,41,99^. We acknowledge that group-level analyses have drawbacks when they are used in isolation to make inferences on the individual-level (the ecologic fallacy)^100^ but we emphasize that they also have clear advantages with exposures that are hard estimate on the individual-level (e.g. nutrients)^101^. They also represent a distinct method of assessment, allowing for more diverse vetting of the relationships being analyzed^102^. Furthermore, group-level studies can help us to avoid the perilous presumption that individual-level factors are the primary determinants of disease and the most effective level for intervention (the individualistic fallacy)^100^. Thus, the approach we used here allows us to consider the possibility that country-level food systems and dietary habits may lead to country-level problems, and these problems could respond to country-level interventions.

### Conclusions

Overall, our findings cannot by themselves provide strong evidence of causation, but in conjunction with the extensive evidence on the level of the individual, they indicate that low Omega-3 intake may be raising MDD and PTB rates in 85% of the countries studied. Furthermore, this analysis reaffirms the importance of considering baseline LC Omega-3 PUFA intakes, sufficiency thresholds, and the endogenous conversion of ALA to EPA, when attempting to estimate associations between LC Omega-3 PUFA intake and health outcomes. In sum, our findings corroborate the links between LC Omega-3 PUFA deficiency, depression, and preterm birth, and they provide a rough estimate of the global scale of these problems. If we want to lower the rates of these expensive and long-lasting consequences, we may have to find safe and sustainable practices for increasing LC Omega-3 PUFA levels in a large majority of the world’s population.

## Supplementary information

Supplementary Information.

## Data Availability

The study data are available in the supplementary information (Table S1). Additional data and details are available from the papers that we referenced as data sources (see below). Country-Level Fatty Acid Intakes. Micha, R. et al. Global, regional, and national consumption levels of dietary fats and oils in 1990 and 2010: a systematic analysis including 266 country-specific nutrition surveys. BMJ (Clinical research ed.) 348, g2272, 10.1136/bmj.g2272 (2014). And the following erratum: Global, regional, and national consumption levels of dietary fats and oils in 1990 and 2010: a systematic analysis including 266 country-specific nutrition surveys. BMJ (Clinical research ed.) 350, h1702, 10.1136/bmj.h1702 (2015). Country-level Depression Prevalences. Ferrari, A. J. et al. Burden of depressive disorders by country, sex, age, and year: findings from the global burden of disease study 2010. PLoS medicine 10, e1001547, 10.1371/journal.pmed.1001547 (2013). Country-level PTB Rates and Income Data. Blencowe, H. et al. National, regional, and worldwide estimates of preterm birth rates in the year 2010 with time trends since 1990 for selected countries: a systematic analysis and implications. Lancet 379, 2162–2172, 10.1016/s0140-6736(12)60820-4 (2012).

## Abbreviations

PTB: Preterm birth
MDD: Major depressive disorder
PUFA: Polyunsaturated fatty acid
ALA: Alpha-linolenic acid – plant based omega-3 PUFA
EPA: Eicosapentaenoic acid
DHA: Docosahexaenoic acid
LC Omega-3 PUFA: Long chain omega-3 polyunsaturated fatty acid (EPA and DHA) – seafood based omega-3 PUFA
Omega-3: A general term referring LC Omega-3 PUFA and its precursors (i.e. ALA, EPA and DHA or plant based and seafood based omega-3 PUFA)
SD: Standard deviation
