# Supplementary material for: Low Omega-3 intake is associated with high rates of depression and preterm birth on the country level
Source: Sci Rep. 2020 Nov 12;10:19749. doi: 10.1038/s41598-020-76552-x (PMC7661496; doi:10.1038/s41598-020-76552-x)
Supplement: Supplementary file 1 — Supplementary Information. [file 41598_2020_76552_MOESM1_ESM.docx]

**Supplementary Information**

**Low Omega-3 intake is associated with high rates of depression and preterm birth on the country level**

Timothy H. Ciesielski^*ab^ and Scott M. Williams^a^

^a^ Department of Population and Quantitative Health Sciences, Case Western Reserve University School of Medicine, Cleveland, Ohio

^b^ Ronin Institute, Montclair, New Jersey

^*^ **Corresponding Author:**

Timothy H. Ciesielski MD MPH ScD

Department of Population and Quantitative Health Sciences

Case Western Reserve University

10900 Euclid Avenue, Cleveland, OH 44106

timothyhciesielski@gmail.com

**Table S1. The Analytic Dataset: Omega-3 Intakes, Preterm Birth Rates, Depression Prevalences, and Country Incomes for 2010**

Countries are listed in order of increasing LC omega3 PUFA intake (males and females combined). Countries with <170 mg/day LC Omega3 PUFA are in brown (n=53 when Afghanistan is included). These countries are at least one standard deviation (380 mg/day) below the putative sufficiency threshold for preterm birth (550 mg/day). Countries in light green have intake norms, that are above putative sufficiency threshold for PTB (>550 mg/day, n=26 when the Maldives are included). Countries in dark green have intake norms that are above putative sufficiency threshold for MDD (>1000 mg/day, n=6 when the Maldives are included).

| **Country ^a^** | **Mean seafood based omega3 intake among adults >20 years old (mg/day) ^a^** | **Mean plant based omega3 intake among adults >20 years old (mg/day) ^a^** | **Estimated total LC Omega3 PUFA among adults >20 years (mg/day) ^b^** | **Country-level preterm birth rate**  **(# preterm births per 100 live births) ^c^** | **Prevalence of MDD (cases per 100 people) ^d^** | **Country Income**  **(0=low 3=high) ^e^** |
| --- | --- | --- | --- | --- | --- | --- |
| Lesotho | 10 | 169 | 35 | 11.9 | 6.3 | 1 |
| Burundi | 25 | 164 | 50 | 11.4 | 6.1 | 0 |
| Honduras | 29 | 153 | 52 | 12.2 | 9.2 | 1 |
| Timor-Leste | 25 | 204 | 56 | 12.1 | 6.0 | 1 |
| Pakistan | 16 | 277 | 58 | 15.8 | 4.8 | 1 |
| Namibia | 23 | 233 | 58 | 14.4 | 5.0 | 2 |
| Swaziland | 18 | 280 | 60 | 13.9 | 5.8 | 1 |
| Rwanda | 18 | 303 | 63 | 9.5 | 7.3 | 0 |
| Botswana | 10 | 387 | 68 | 15.1 | 7.4 | 2 |
| Eritrea | 11 | 406 | 72 | 12.2 | 6.6 | 0 |
| Kenya | 41 | 217 | 74 | 12.3 | 5.2 | 0 |
| Ethiopia | 48 | 240 | 84 | 10.1 | 3.6 | 0 |
| Niger | 28 | 389 | 86 | 9.4 | 4.4 | 0 |
| Azerbaijan | 37 | 342 | 88 | 8.5 | 4.2 | 2 |
| Nepal | 20 | 468 | 90 | 14.0 | 3.0 | 0 |
| Bhutan | 23 | 460 | 92 | 10.2 | 3.8 | 1 |
| Sudan | 17 | 526 | 96 | 13.2 | 7.1 | 1 |
| Burkina Faso | 22 | 498 | 97 | 10.9 | 4.0 | 0 |
| Bolivia | 16 | 545 | 98 | 9.0 | 3.9 | 1 |
| Mongolia | 15 | 557 | 99 | 13.5 | 4.8 | 1 |
| Somalia | 48 | 337 | 99 | 12.0 | 6.3 | 0 |
| Zimbabwe | 5 | 630 | 100 | 16.6 | 6.5 | 0 |
| Kyrgyzstan | 44 | 397 | 104 | 10.4 | 4.8 | 0 |
| Yemen | 52 | 362 | 106 | 13.2 | 7.1 | 1 |
| Mexico | 39 | 464 | 109 | 7.3 | 3.0 | 2 |
| Djibouti | 13 | 640 | 109 | 11.9 | 6.7 | 1 |
| Armenia | 41 | 466 | 111 | 11.0 | 5.0 | 1 |
| Malawi | 52 | 393 | 111 | 18.1 | 5.8 | 0 |
| Bangladesh | 47 | 431 | 112 | 14.0 | 3.4 | 0 |
| El Salvador | 45 | 453 | 113 | 12.8 | 5.4 | 1 |
| India | 31 | 552 | 114 | 13.0 | 4.1 | 1 |
| South Africa | 14 | 683 | 116 | 8.0 | 4.6 | 2 |
| Nicaragua | 37 | 542 | 118 | 9.3 | 5.2 | 1 |
| Montenegro | 75 | 293 | 119 | 9.2 | 6.0 | 2 |
| Congo, Democratic Republic | 62 | 382 | 119 | 11.9 | 5.8 | 0 |
| Tanzania, United Republic | 69 | 339 | 120 | 11.4 | 6.4 | 0 |
| Liberia | 51 | 461 | 120 | 13.9 | 4.6 | 0 |
| Guatemala | 21 | 703 | 126 | 7.7 | 5.4 | 1 |
| Mozambique | 25 | 679 | 127 | 16.4 | 4.6 | 0 |
| Afghanistan | 30 | 665 | 130 | 11.5 | 22.5 | 0 |
| Uzbekistan | 15 | 773 | 131 | 8.7 | 5.5 | 1 |
| Albania | 47 | 563 | 131 | 9.0 | 5.2 | 2 |
| Belize | 73 | 490 | 147 | 10.4 | 5.4 | 1 |
| Costa Rica | 54 | 621 | 147 | 13.6 | 4.7 | 2 |
| Turkmenistan | 67 | 570 | 153 | 9.8 | 6.2 | 1 |
| Benin | 75 | 520 | 153 | 10.6 | 3.9 | 0 |
| Hungary | 72 | 562 | 156 | 8.6 | 3.3 | 3 |
| Saudi Arabia | 58 | 656 | 156 | 6.0 | 5.9 | 3 |
| Zambia | 68 | 612 | 160 | 12.9 | 5.8 | 1 |
| Madagascar | 72 | 590 | 161 | 14.2 | 5.1 | 0 |
| Haiti | 36 | 841 | 162 | 14.1 | 4.8 | 0 |
| Egypt | 77 | 568 | 162 | 7.3 | 5.3 | 1 |
| Georgia | 112 | 379 | 169 | 8.8 | 4.9 | 1 |
| Colombia | 94 | 510 | 171 | 8.8 | 6.3 | 2 |
| Cote d'Ivoire (Ivory Coast) | 140 | 205 | 171 | 14.0 | 5.1 | 1 |
| Trinidad and Tobago | 22 | 996 | 171 | 8.1 | 5.5 | 3 |
| Guinea-Bissau | 10 | 1079 | 172 | 11.2 | 3.9 | 0 |
| Mali | 76 | 667 | 176 | 11.6 | 5.7 | 0 |
| Togo | 94 | 566 | 179 | 13.3 | 4.5 | 0 |
| Singapore | 49 | 887 | 182 | 11.5 | 5.7 | 3 |
| Iraq | 44 | 922 | 182 | 6.5 | 4.5 | 1 |
| Bosnia and Herzegovina | 67 | 790 | 186 | 7.9 | 3.6 | 2 |
| Syrian Arab Republic | 17 | 1134 | 187 | 10.9 | 7.0 | 1 |
| Cuba | 62 | 836 | 187 | 6.4 | 5.8 | 2 |
| Chad | 72 | 771 | 188 | 13.1 | 5.2 | 0 |
| Nigeria | 64 | 834 | 189 | 12.2 | 3.7 | 1 |
| Tajikistan | 19 | 1134 | 189 | 10.7 | 5.0 | 0 |
| Kazakhstan | 59 | 870 | 190 | 8.8 | 4.2 | 2 |
| Ecuador | 36 | 1027 | 190 | 5.1 | 4.4 | 2 |
| Ireland | 97 | 626 | 191 | 6.4 | 4.1 | 3 |
| Uganda | 113 | 557 | 197 | 13.6 | 6.4 | 0 |
| Moldova | 108 | 613 | 200 | 11.9 | 4.4 | 1 |
| Serbia | 57 | 1020 | 210 | 6.7 | 4.0 | 2 |
| Cameroon | 142 | 503 | 217 | 12.6 | 4.4 | 1 |
| Saint Vincent and the Grenadines | 114 | 702 | 219 | 11.8 | 5.4 | 2 |
| Oman | 45 | 1181 | 222 | 14.3 | 5.3 | 3 |
| United Arab Emirate | 78 | 962 | 222 | 7.6 | 8.1 | 3 |
| Dominica | 154 | 485 | 227 | 11.9 | 5.4 | 2 |
| Iran, Islamic Republic | 50 | 1195 | 229 | 12.9 | 7.0 | 2 |
| Bahamas | 169 | 405 | 230 | 9.5 | 5.4 | 3 |
| Panama | 130 | 667 | 230 | 8.1 | 4.7 | 2 |
| Israel | 232 | 2 | 232 | 8.0 | 4.6 | 3 |
| Guinea | 92 | 968 | 237 | 13.9 | 4.5 | 0 |
| Argentina | 48 | 1304 | 244 | 8.0 | 5.2 | 2 |
| Slovakia | 59 | 1253 | 247 | 6.3 | 3.6 | 3 |
| Libyan Arab Jamahiriya | 60 | 1265 | 250 | 8.3 | 9.3 | 2 |
| Kuwait | 76 | 1159 | 250 | 10.6 | 7.5 | 3 |
| Morocco | 63 | 1283 | 255 | 6.7 | 6.9 | 1 |
| Macedonia, Former Yugoslav Republic | 59 | 1316 | 256 | 6.8 | 5.2 | 2 |
| Belarus | 116 | 944 | 258 | 4.1 | 6.6 | 2 |
| Jordan | 47 | 1410 | 259 | 14.4 | 7.7 | 2 |
| Romania | 61 | 1335 | 261 | 7.3 | 4.3 | 2 |
| Comoros | 243 | 126 | 262 | 16.7 | 5.8 | 0 |
| Guyana | 228 | 249 | 265 | 13.2 | 6.3 | 1 |
| Mauritania | 142 | 832 | 267 | 15.4 | 5.0 | 1 |
| Central African Republic | 38 | 1529 | 267 | 12.6 | 5.7 | 0 |
| Peru | 147 | 807 | 268 | 7.3 | 4.9 | 2 |
| Greece | 209 | 400 | 269 | 6.6 | 4.9 | 3 |
| Bahrain | 52 | 1451 | 270 | 14.0 | 8.6 | 3 |
| Venezuela, Bolivarian Republic | 121 | 994 | 270 | 8.1 | 5.1 | 2 |
| Slovenia | 69 | 1355 | 272 | 7.5 | 4.5 | 3 |
| Qatar | 50 | 1505 | 276 | 10.5 | 8.0 | 3 |
| Netherlands | 180 | 640 | 276 | 8.0 | 8.0 | 3 |
| Uruguay | 69 | 1384 | 277 | 10.1 | 4.7 | 2 |
| Sierra Leone | 205 | 487 | 278 | 10.0 | 5.7 | 0 |
| Saint Lucia | 263 | 129 | 282 | 11.1 | 4.3 | 2 |
| Brunei Darussalam | 239 | 290 | 283 | 12.1 | 4.4 | 3 |
| Switzerland | 212 | 508 | 288 | 7.4 | 6.2 | 3 |
| Dominican Republic | 54 | 1566 | 289 | 10.8 | 4.5 | 2 |
| Luxembourg | 233 | 390 | 292 | 8.1 | 6.6 | 3 |
| Poland | 107 | 1250 | 295 | 6.7 | 3.9 | 3 |
| Austria | 184 | 738 | 295 | 10.9 | 5.0 | 3 |
| Lebanon | 8 | 1918 | 296 | 7.9 | 5.3 | 2 |
| Paraguay | 62 | 1575 | 298 | 7.8 | 6.4 | 1 |
| Equatorial Guinea | 94 | 1391 | 303 | 16.5 | 7.1 | 3 |
| Cape Verde | 120 | 1232 | 305 | 11.2 | 5.0 | 1 |
| Democratic People's Republic of Korea | 23 | 1899 | 308 | 10.7 | 2.6 | 0 |
| Congo | 148 | 1067 | 308 | 16.7 | 6.5 | 1 |
| Czech Republic | 113 | 1364 | 318 | 7.3 | 3.2 | 3 |
| Sao Tome and Principe | 200 | 792 | 319 | 10.5 | 6.5 | 1 |
| Brazil | 57 | 1747 | 319 | 9.2 | 5.5 | 2 |
| Cyprus | 213 | 741 | 324 | 14.7 | 5.8 | 3 |
| Croatia | 145 | 1196 | 324 | 5.5 | 7.1 | 3 |
| Russian Federation | 180 | 992 | 329 | 7.0 | 6.5 | 2 |
| Suriname | 133 | 1308 | 329 | 8.8 | 5.4 | 2 |
| Bulgaria | 119 | 1444 | 336 | 7.5 | 4.6 | 2 |
| Gabon | 199 | 925 | 338 | 16.3 | 7.2 | 2 |
| Ukraine | 180 | 1057 | 339 | 6.5 | 3.9 | 1 |
| Malta | 279 | 402 | 339 | 6.4 | 6.6 | 3 |
| Papua New Guinea | 294 | 303 | 339 | 6.5 | 5.0 | 1 |
| Ghana | 268 | 569 | 353 | 14.5 | 4.4 | 1 |
| Algeria | 42 | 2180 | 369 | 7.4 | 7.3 | 2 |
| Belgium | 281 | 590 | 370 | 7.9 | 4.0 | 3 |
| United States of America | 141 | 1527 | 370 | 12.0 | 4.5 | 3 |
| Estonia | 188 | 1217 | 371 | 5.7 | 6.8 | 3 |
| Italy | 281 | 713 | 388 | 6.5 | 4.8 | 3 |
| Gambia | 196 | 1336 | 396 | 14.0 | 5.1 | 0 |
| Vanuatu | 353 | 333 | 403 | 12.9 | 4.4 | 1 |
| Canada | 92 | 2085 | 405 | 7.8 | 4.4 | 3 |
| Grenada | 254 | 1069 | 414 | 10.3 | 5.4 | 2 |
| Angola | 87 | 2195 | 416 | 12.5 | 5.0 | 1 |
| Tunisia | 84 | 2215 | 416 | 8.9 | 7.1 | 2 |
| Lithuania | 230 | 1253 | 418 | 5.7 | 4.8 | 2 |
| Australia | 286 | 914 | 423 | 7.6 | 3.1 | 3 |
| Micronesia, Federated States | 368 | 374 | 424 | 10.5 | 4.4 | 1 |
| Solomon Islands | 412 | 102 | 427 | 12.4 | 3.4 | 1 |
| Latvia | 180 | 1665 | 430 | 5.3 | 6.2 | 2 |
| Germany | 226 | 1404 | 437 | 9.2 | 4.9 | 3 |
| Marshall Islands | 388 | 430 | 453 | 11.5 | 5.7 | 1 |
| Antigua and Barbuda | 310 | 1059 | 469 | 5.8 | 7.1 | 2 |
| Fiji | 321 | 1258 | 510 | 9.9 | 3.5 | 1 |
| Tonga | 441 | 464 | 511 | 7.5 | 4.4 | 1 |
| France | 404 | 736 | 514 | 6.7 | 4.8 | 3 |
| Lao People's Democratic Republic | 489 | 223 | 522 | 10.8 | 5.8 | 1 |
| Kiribati | 496 | 183 | 523 | 9.6 | 5.7 | 1 |
| China | 37 | 3266 | 527 | 7.1 | 3.0 | 2 |
| New Zealand | 374 | 1041 | 530 | 7.6 | 4.7 | 3 |
| Samoa | 521 | 152 | 544 | 5.5 | 4.4 | 1 |
| Senegal | 229 | 2183 | 556 | 9.7 | 4.5 | 1 |
| Chile | 407 | 1230 | 592 | 7.1 | 4.0 | 2 |
| Mauritius | 395 | 1326 | 594 | 12.6 | 5.1 | 2 |
| Turkey | 377 | 1501 | 602 | 12.0 | 6.7 | 2 |
| Sweden | 400 | 1355 | 603 | 5.9 | 4.8 | 3 |
| Philippines | 590 | 131 | 610 | 14.9 | 5.2 | 1 |
| Viet Nam | 574 | 278 | 616 | 9.4 | 4.0 | 1 |
| Portugal | 582 | 604 | 673 | 7.7 | 4.3 | 3 |
| United Kingdom | 318 | 2414 | 680 | 7.8 | 3.1 | 3 |
| Myanmar | 620 | 593 | 709 | 12.4 | 5.2 | 0 |
| Cambodia | 680 | 215 | 712 | 10.5 | 4.9 | 0 |
| Sri Lanka | 720 | 106 | 736 | 10.7 | 4.7 | 1 |
| Norway | 596 | 1076 | 757 | 6.0 | 5.9 | 3 |
| Spain | 647 | 874 | 778 | 7.4 | 4.3 | 3 |
| Indonesia | 755 | 256 | 793 | 15.5 | 4.9 | 1 |
| Finland | 511 | 2014 | 813 | 5.5 | 6.0 | 3 |
| Republic of Korea | 708 | 883 | 840 | 9.2 | 2.5 | 3 |
| Thailand | 824 | 506 | 900 | 12.0 | 5.2 | 2 |
| Japan | 718 | 1224 | 902 | 5.9 | 2.5 | 3 |
| Jamaica | 82 | 5542 | 913 | 10.2 | 4.8 | 2 |
| Malaysia | 988 | 387 | 1046 | 12.3 | 5.6 | 2 |
| Denmark | 1225 | 300 | 1270 | 6.7 | 5.1 | 3 |
| Seychelles | 1291 | 637 | 1387 | 11.6 | 5.2 | 2 |
| Iceland | 1229 | 1271 | 1420 | 6.5 | 4.7 | 3 |
| Barbados | 1986 | 165 | 2011 | 8.9 | 5.3 | 3 |
| Maldives | 3886 | 215 | 3918 | 7.9 | 5.1 | 2 |

^a^ Information from ^1,2^ (reference 2 provides a correction for the data supplied in reference 1)

^b^ Total LC omega-3 PUFA = seafood based + (plant based x 0.15)

^c^ Information from ^3^

^d^ Information from ^4^

^e^ Gross National Income is an ordinal variable (0=low, 3=high) – information from ^3^

**Figure S1. Distribution of LC Omega-3 PUFA Dietary Intakes**

LC Omega-3 PUFA = seafood based intake + (plant based intake x 0.15)

The Maldives are over 9 SDs above the mean at 3918 mg/day*. Figure made with SAS 9.4 (https://www.sas.com/en_us/home.html SAS Institute, Cary NC)*


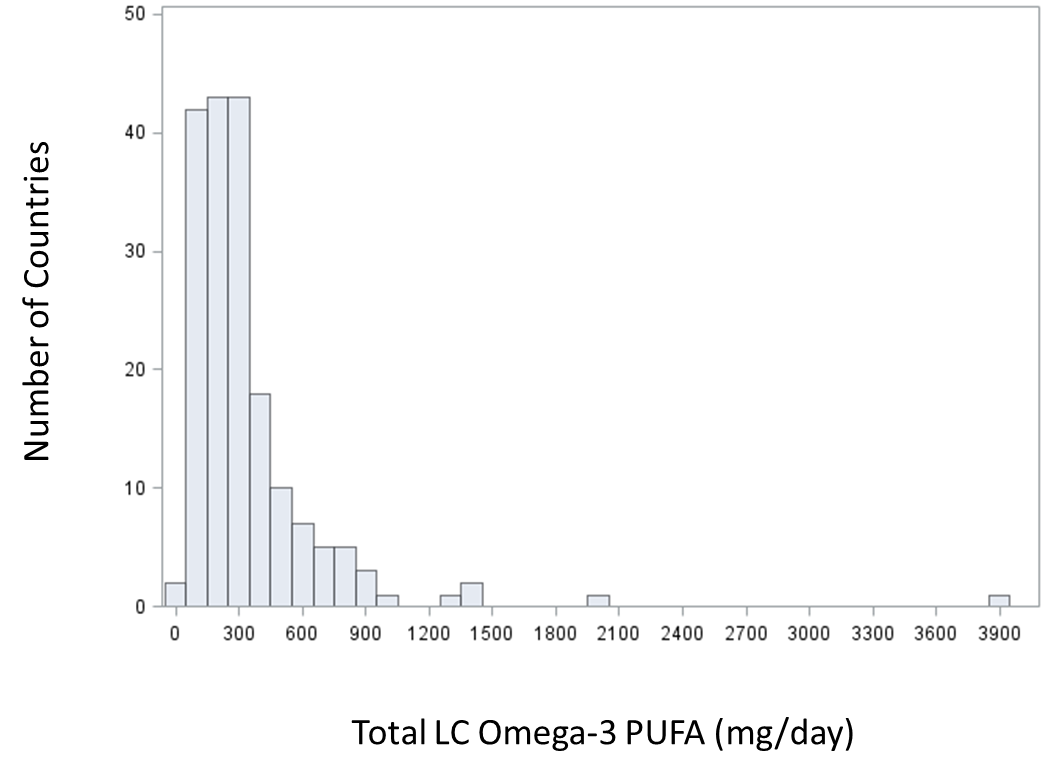


**Figure S2. Scatterplot of LC Omega-3 PUFA Intake by Country Income** LC Omega-3 PUFA = seafood based intake + (plant based intake x 0.15) Country income was recorded as an ordinal variable with 4 ranks (Gross National Income: 4 = high per capita income, 0 = low per capita income). One omega 3 outlier was omitted (Maldives: LC Omega-3 PUFA = 3918 mg/day, GNI = 2). The Spearman Rank Correlation was 0.44 and this was unchanged when the Maldives were included. *Figure made with SAS 9.4 (https://www.sas.com/en_us/home.html SAS Institute, Cary NC)*


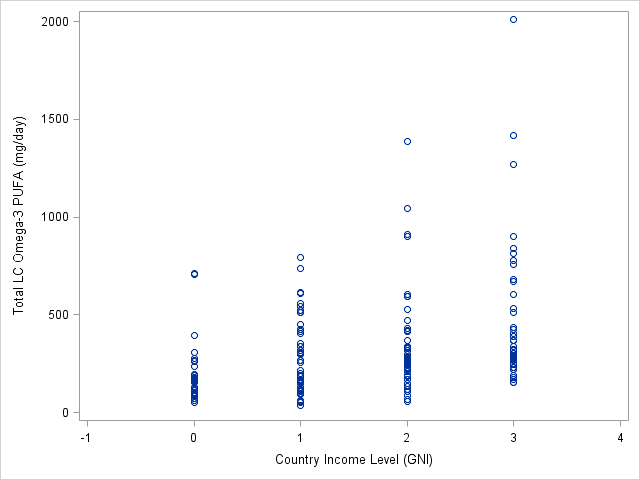


**Figure S3: Penalized Splines Modeling the Seafood Based and Plant Based Omega 3 Separately**

**
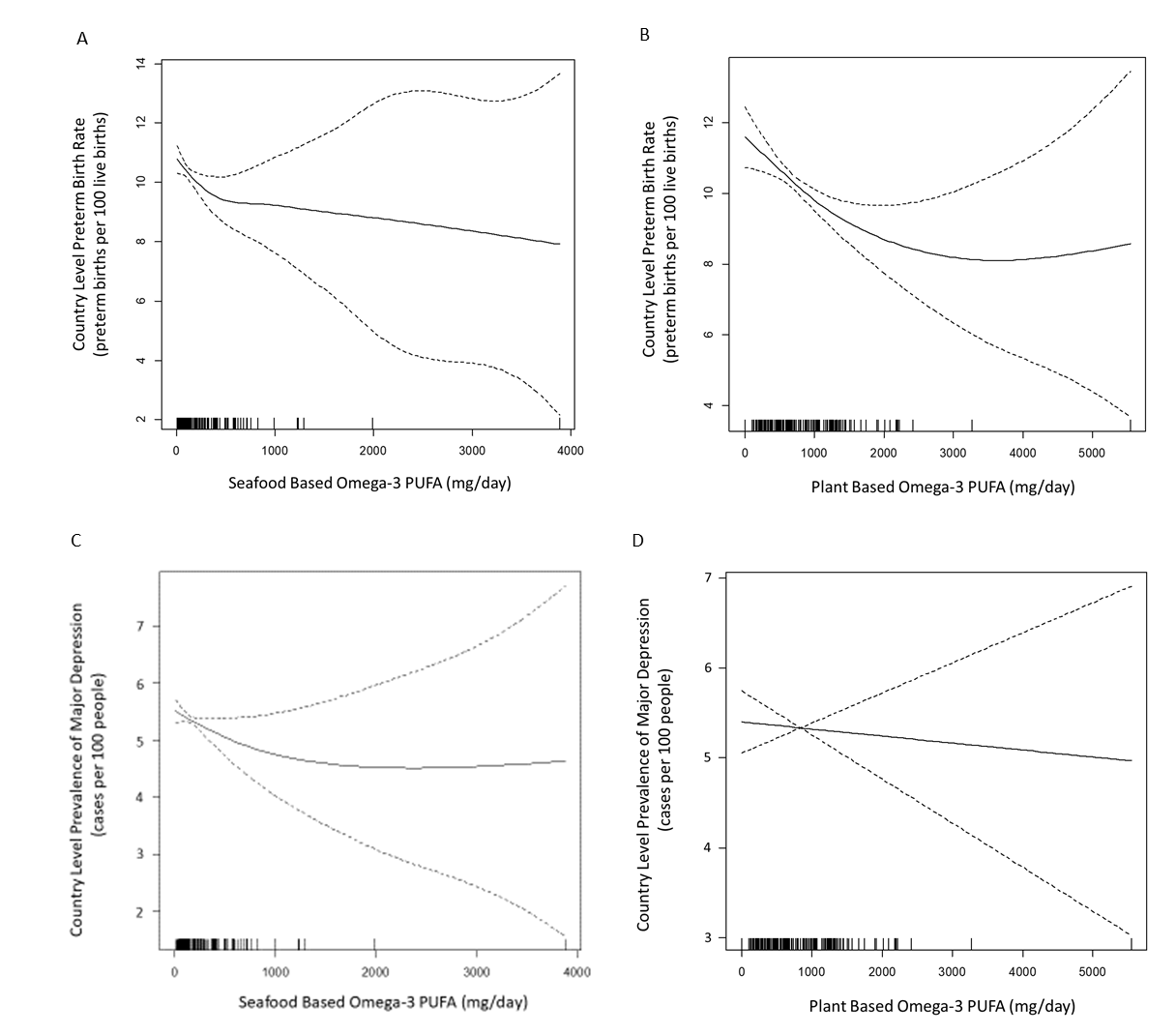
**

Panel A: Seafood based omega-3 intake norms are on the x axis and preterm birth rates are on the y axis. A putative threshold is evident around 500-600 mg/day. The estimated spline is most certain in sections of the exposure distribution with the greatest data density. Each vertical dash below the spline represents a single country, and this displays information on data density in different regions of the exposure distribution.

Panel B: Plant based omega-3 intake norms are on the x axis and preterm birth rates are on the y axis. A putative threshold is evident at about 3000-4000 mg/day.

Panel C: Seafood based omega-3 intake norms are on the x axis and MDD prevalences are on the y axis. A putative threshold is evident at about 900-1100 mg/day

Panel B: Plant based omega-3 intake norms are on the x axis and MDD prevalences are on the y axis. No threshold is evident.

*Figure made with R 3.5.0 (https://www.r-project.org/)*

*Because both plant based and seafood based omega3 intake both contribute to internal levels of LC Omega3 PUFA it is difficult to interpret their relationships with these outcomes when they are considered in isolation. As independent variables they both represent an incomplete measurement of our exposure of interest. Having said this, by separating these exposure components we can roughly assess the conversion rate that we use in our models. For PTB the threshold detected in the seafood based analysis (400-550 mg/day) is approximately 15% of the threshold observed in the plant based analysis (2,600-3,600 mg/day). This is what we would expect to see if conversion to LC Omega3 PUFA was driving the pattern in the plant based analysis. For MDD there was no threshold detected in the plant based analysis, and this is to be expected if the 15% conversion rate is approximately accurate. Stated differently, the seafood based threshold is estimated at 850-1100 mg/day for MDD, therefore the plant based threshold would be expected to be found around 5,600-7,300 mg/day, and we have no exposure data in this range. Because seafood and plant based Omega-3 are isolated both contribute to total LC Omega-3 PUFA exposure, they only serve as rough proxies for exposure of interest. Thus there is limited precision in these splines and threshold locations, but this is what we would expect to see if total LC Omega-3 PUFA was driving the associations in our main analyses.*

**Figure S4. Penalized Splines Modeling the Relationship between LC Omega3 PUFA and PTB Using Alternative Conversion Rates for Plant Based Precursors**

Panels A-F: Country-level LC Omega3 PUFA intakes are on the x axis and country-level preterm birth rates are on the y axis. The assumed ALA to EPA/DHA conversion rate is listed below each panel. *Figure made with R 3.5.0 (https://www.r-project.org/)*

**
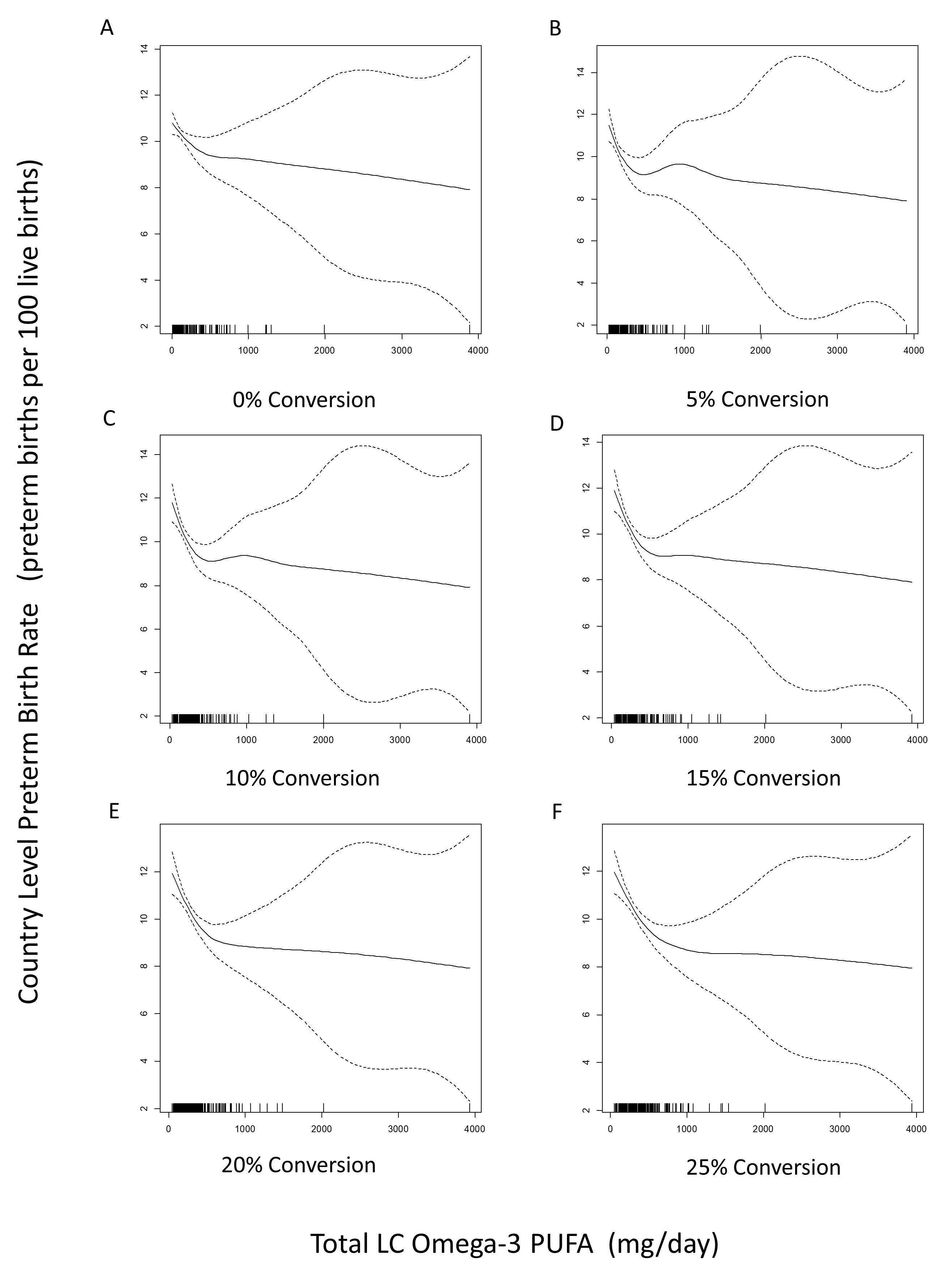
**

**Figure S5. Penalized Splines Modeling the Relationship between LC Omega3 PUFA and MDD Using Alternative Conversion Rates for Plant Based Precursors**

Panels A-F: Country-level LC Omega3 PUFA intakes are on the x axis and country-level MDD prevalences are on the y axis. The assumed ALA to EPA/DHA conversion rate is listed below each panel. *Figure made with R 3.5.0 (https://www.r-project.org/)*

**
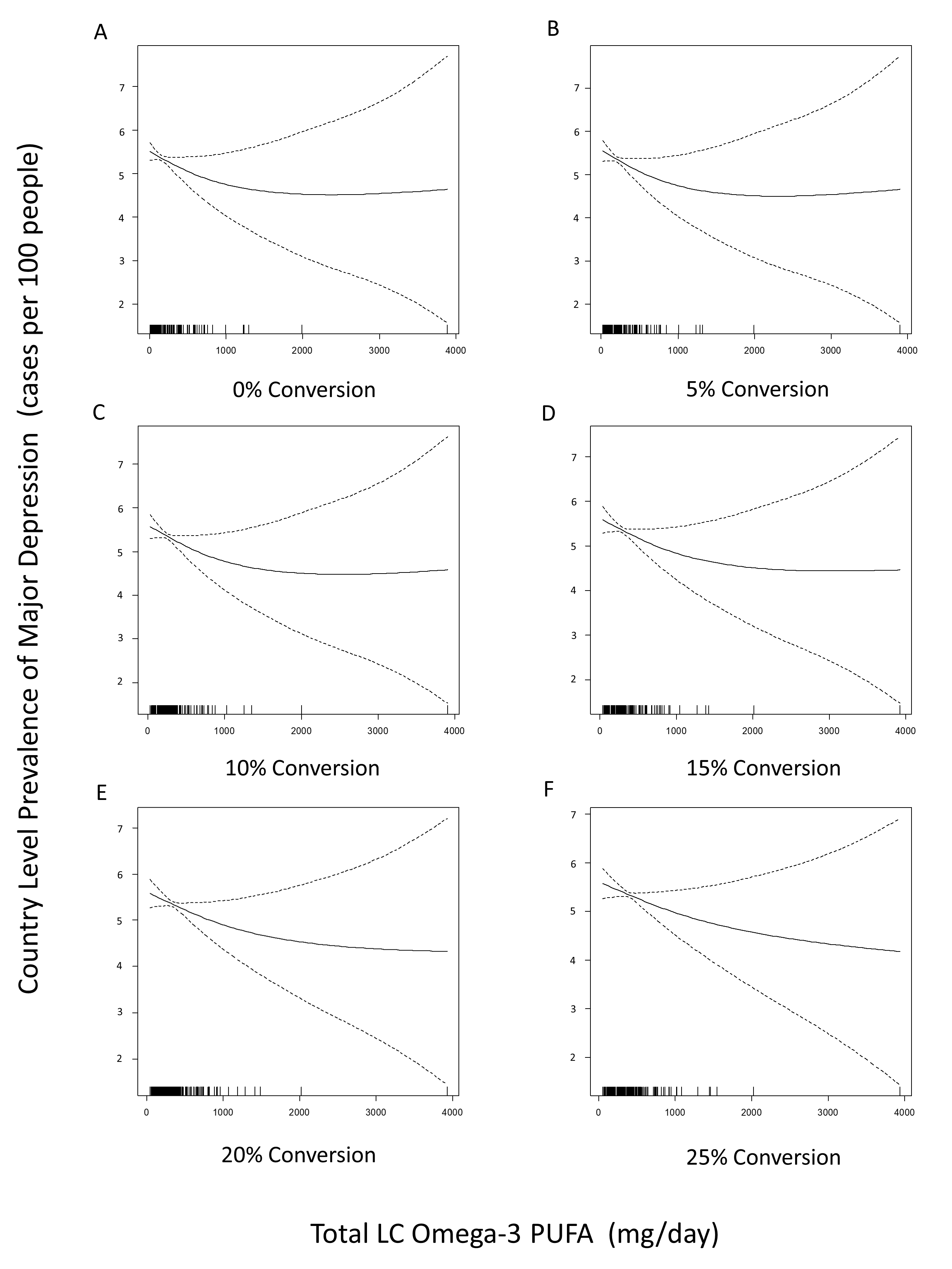
**

**Figure S6. Estimating the omega3 MDD association with minimal bias**

Several plausible Directed Acyclic Graphs (DAGs) yield different conclusions about what adjustments could reduce bias. *Figure made with Microsoft PowerPoint 2019 (https://www.microsoft.com/en-us/).*

**
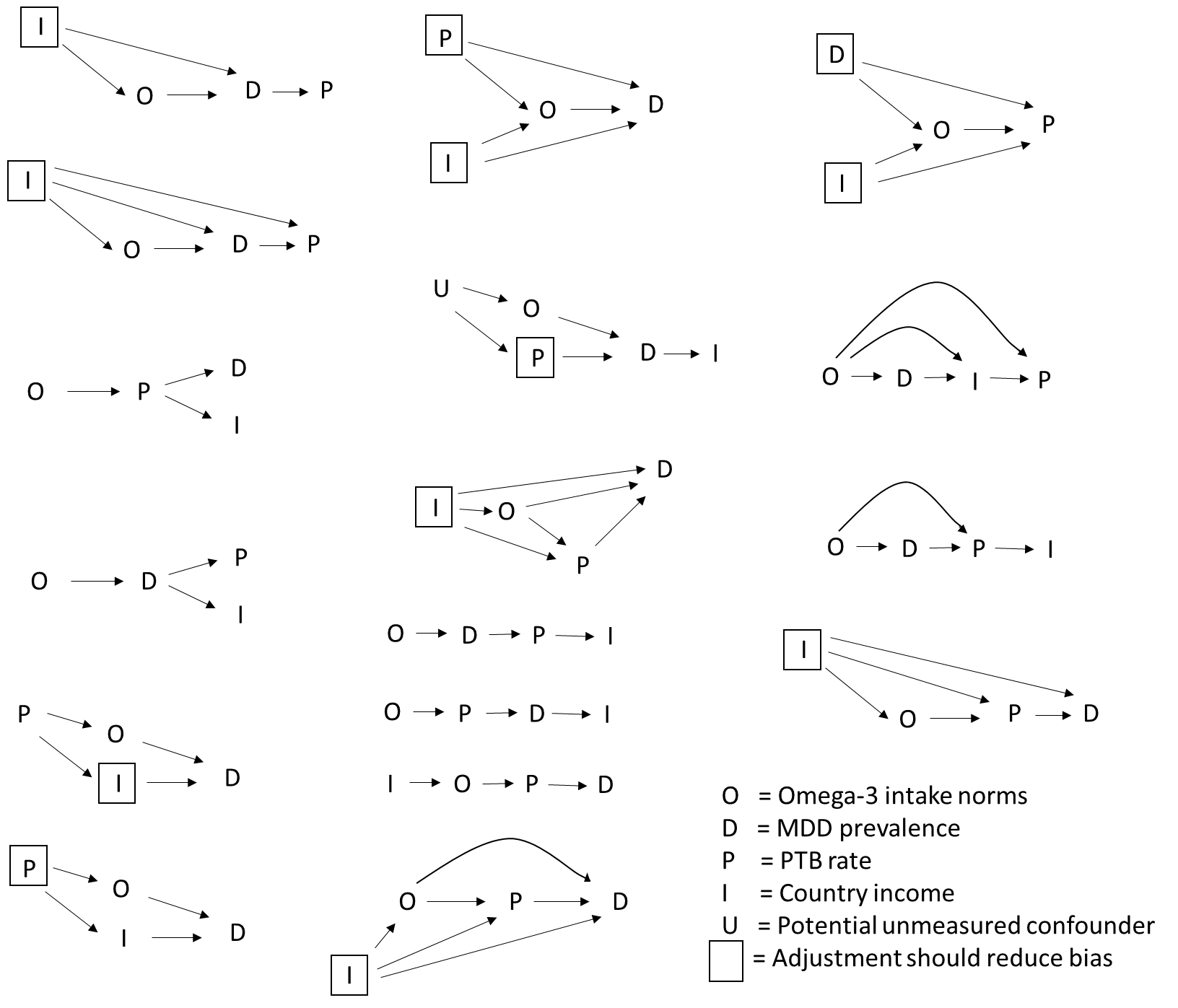
**

**References for the Supplementary Information**

1. Micha, R. *et al.* Global, regional, and national consumption levels of dietary fats and oils in 1990 and 2010: a systematic analysis including 266 country-specific nutrition surveys. *BMJ (Clinical research ed.)* **348**, g2272 (2014).

2. Global, regional, and national consumption levels of dietary fats and oils in 1990 and 2010: a systematic analysis including 266 country-specific nutrition surveys. *BMJ (Clinical research ed.)* **350**, h1702 (2015).

3. Blencowe, H. *et al.* National, regional, and worldwide estimates of preterm birth rates in the year 2010 with time trends since 1990 for selected countries: a systematic analysis and implications. *Lancet* **379**, 2162–72 (2012).

4. Ferrari, A. J. *et al.* Burden of depressive disorders by country, sex, age, and year: findings from the global burden of disease study 2010. *PLoS medicine* **10**, e1001547 (2013).
